# Mandibular Advancement Device Therapy in Japanese Rugby Athletes with Poor Sleep Quality and Obstructive Sleep Apnea

**DOI:** 10.3390/life12091299

**Published:** 2022-08-24

**Authors:** Hiroshi Suzuki, Toshiyuki Nakayama, Arisa Sawa, Tatsuo Yagi, Yoshihiro Iwata, Hiroki Takeuchi, Miho Motoyoshi, Chin-Moi Chow, Osamu Komiyama

**Affiliations:** 1 Department of Oral Function and Fixed Prosthodontics, Nihon University School of Dentistry at Matsudo, Chiba 271-8587, Japan; 2 Department of Physical Reaction, Tokai University School of Physical Education, Hiratsuka-shi 259-1292, Japan; 3 Sleep Research Group, Charles Perkins Centre, University of Sydney, Sydney 2006, Australia; 4 Sydney School of Health Sciences, Faculty of Medicine and Health, University of Sydney, Sydney 2006, Australia

**Keywords:** motion perception, oral appliance therapy, prevalence, reaction time, obstructive sleep apnea, sleep quality

## Abstract

Obstructive sleep apnea (OSA) may contribute to poor sleep quality. This study assessed subjective sleep quality, the Respiratory Event Index (REI), reaction times, and the therapeutic effects of a custom-made mandibular advancement device (MAD) in male Japanese elite rugby athletes. The Pittsburgh Sleep Quality Index (PSQI), Epworth Sleepiness Scale (ESS), and level III sleep test (REI and minimum oxygen saturation [SpO_2_ _min_]) were used to evaluate sleep quality. MAD therapy was used daily for 3 weeks. A telephone-based reaction time test of kinetic vision (the ability to identify moving objects) was recorded within 15 min of waking and over 5 days of pre- and post-MAD therapy. Differences in variables were evaluated using paired *t*-tests. Of the 42 players (mean age, 26.3 ± 3.7 years; mean body mass index, 28.7 ± 3.2 kg/m^2^) included in this study, 29 (69.0%) had poor sleep quality (PSQI > 5.5), and 27 were diagnosed with OSA (64.3%) (mild = 16/moderate = 9/severe = 2). Six were treated with MAD therapy, which significantly improved the REI (*p* < 0.01), SpO_2_ _min_ (*p* < 0.001), ESS score (*p* < 0.001), reaction times (*p* < 0.01), and sleep quality. A significant reduction in reaction times suggests that OSA treatment can improve kinetic vision. Future studies should systematically evaluate the impact of sleep-disordered breathing on kinetic vision in athletes.

## 1. Introduction

Obstructive sleep apnea (OSA), a condition arising from repeated events of airway closure and opening during sleep, is a major but treatable cause of sleep-disordered breathing (SDB), which is reported to affect nearly 1 billion people worldwide [1]. SDB is a current medical and social problem because it can lead to future cardiovascular disease [2,3], workplace errors and injuries, traffic accidents, and other problems [1,4].

The prevalence of OSA is conservatively estimated to be 3% among women and 10% among men aged 30–49 years and 9% among women and 17% among men aged 50–70 years [5]. Several studies have associated daytime sleepiness, depression, anxiety, and locomotive syndrome with OSA in Japanese patients [6,7,8]. Sudden cardiac death in athletes has also been linked to cardiac arrhythmia caused by OSA [9,10].

Continuous positive airway pressure (CPAP) is a therapeutic option for SDB that can reduce excessive daytime sleepiness and improve cognitive function [11,12] as well as reduce the risk of cardiovascular disease associated with SDB [13,14,15]. The use of an oral appliance, such as a mandibular advancement device (MAD), has recently been introduced to treat patients with mild-to-moderate OSA [16]. The demand for oral appliances has increased with an increasing number of people refusing CPAP or requesting MAD (e.g., for overnight business travel) [17,18].

In studies of football players, the prevalence of SDB was unexpectedly high at 14.0–86.5% [19,20], compared with a prevalence of 7.9–17.0% in adults in their 30s in the general population [7,8,9]. In a study of golfers, SDB reduced performance, which was restored with CPAP therapy [21]. In a study of judo players [22], CPAP therapy reduced excessive daytime sleepiness. These results suggest that SDB is prevalent among athletes and that CPAP therapy is recommended to improve performance. Although CPAP is the gold standard of treatment for OSA, oral appliance treatment is a common practice in Japan, where it is considered a better alternative to CPAP when polysomnography results show that CPAP is not suitable.

In the case of rugby union athletes, we expected the prevalence of OSA to be high, especially in those in the heavier weight classes. We previously reported that the prevalence rate of OSA in Japanese rugby athletes was 86.5%. Potential contributing factors included a large neck circumference and the characteristic facial morphology of Japanese individuals [23]. Another report suggested that both SDB and excessive daytime sleepiness are common in elite rugby union athletes [20]. An accurate diagnosis of OSA is essential for treatment, recovery, and athletic performance. Although the effectiveness of oral appliance treatment in improving OSA in athletes has been recognized, studies of the association with sports performance are lacking, and further investigation is necessary.

SDB affects vigilance and judgment ability in athletes; however, data are scarce. Reaction time affects sports performance, particularly in activities requiring fast reactions and coordinated movements [24]. Several reaction time tests (e.g., the Oxford Sleep Resistance test, Multiple Unprepared Reaction Time test) have been used to monitor improvements in vigilance following sleep apnea treatment in professional drivers [25]. A reduced reaction time after CPAP therapy has also been reported as a reliable indicator of treatment efficacy in patients with OSA [21]. Reaction time is also used to evaluate sports performance in response to sleep deprivation [26].

No reports have indicated improvements in sports performance in athletes undergoing MAD therapy. In this study, we evaluated the effect of MAD therapy on the reaction time of rugby athletes, focusing on kinetic vision (the ability to identify moving objects). Specifically, we report on sleep quality, the Respiratory Event Index (REI), daytime sleepiness, and reaction times of rugby union athletes as well as the outcomes of MAD therapy in a subgroup of players.

## 2. Materials and Methods

### 2.1. Participants

In this observational study, 42 Japanese male professional rugby union athletes were examined. The team received routine dental care at the Nihon University School of Dentistry at Matsudo (Chiba, Japan). Dental services included dental checkups and treatment as well as the provision of custom-made mouthguards in May before the start of the season. Consent was obtained for participation in this study during the checkup. 

Table 1 shows the patient characteristics, including the mean (±standard deviation) age, height, weight, body mass index (BMI), and neck circumference. The health of the team athletes was generally good, and none of the athletes were taking medications. Dental evaluation of all the athletes showed normal occlusion, without subjective or objective abnormalities in the stomatognathic system, and no past or present disorders of the maxillomandibular joint or motor dysfunction of the trunk or limbs.

### 2.2. Pittsburgh Sleep Quality Index

The Pittsburgh Sleep Quality Index (PSQI) comprises 19 self-rated questions. The athletes were asked about their bedtime, sleep latency, and wake-up time. Sleep duration and efficiency were calculated. The subjective sleep quality was assessed using the global PSQI score. In the Japanese version, a global PSQI score > 5.5 indicates poor sleep quality [27].

### 2.3. Evaluation of Daytime Sleepiness

Subjective sleepiness was assessed using the Japanese version of the Epworth Sleepiness Scale (ESS), a simple questionnaire measuring the general level of daytime sleepiness or average sleep propensity experienced by an individual. This index produces a global score ranging from 0 to 24, where a higher score indicates more daytime sleepiness. An ESS score >10 indicates excessive daytime sleepiness [28]. The ESS score is comparable to other daytime sleepiness tests, such as the multiple sleep latency test, which is a valid and reliable measure of objective sleepiness.

### 2.4. Out-of-Center Sleep Test

Athletes were instructed not to drink alcohol, take drugs that may affect the test results, or perform other activities that affect sleep (e.g., excessive exercise without prior training or any all-night activity) the day before the test. Level III portable monitors (SAS-2100; Teijin Home Healthcare, Tokyo, Japan) compliant with American Academy of Sleep Medicine standards were loaned to athletes after they received information on the relevance of the test and instructions on how to operate the device. Athletes wore the device at night for a minimum of 6 h while sleeping in their homes. 

The minimum oxygen saturation (SpO_2 min_) was recorded. The devices were collected on the day after the test. Laboratory technicians, who were blinded to the athletes’ background, downloaded the data onto personal computers, analyzed the monitoring records using dedicated software (QP-021W version 01-10; Nihon Kohden, Tokyo, Japan), and visualized the data.

The REI was calculated as the total number of respiratory events divided by the monitoring time. OSA severity was classified as mild (5 ≤ REI < 15), moderate (15 ≤ REI < 30), or severe (REI ≥ 30).

### 2.5. MAD Therapy

Athletes were informed of the outcome of the sleep apnea test following completion of the assessments. Those who met the criteria for MAD therapy were offered treatment. In Japan, MAD therapy is approved by the National Health Insurance system for patients with REI > 5, whereas CPAP treatment requires polysomnography (PSG) monitoring when the REI is <30 (PSG is not required if the REI is ≥40). Thus, MAD was offered to the athletes, and a level III portable sleep monitor was used in this study. 

Of the 27 athletes diagnosed with OSA, six athletes (forward position, *n* = 3; back position, *n* = 3) (mean age, 27.2 ± 4.7 years; mean height, 173.3 ± 5.6 cm; mean weight, 94.2 ± 13.4 kg; mean BMI, 31.4 ± 4.5 kg/m^2^; mean neck circumference, 42.1 ± 2.0 cm) requested and received a monoblock MAD at the outpatient sleep clinic of the Nihon University School of Dentistry at Matsudo Hospital. In a pre-post study, this subgroup of athletes underwent MAD therapy for 3 weeks with sleep, ESS, and reaction time assessments, when they were already accustomed to wearing the MAD.

### 2.6. Reaction Time Test of Kinetic Vision

Reaction time was measured 15 min after awakening to evaluate the kinetic vision of the six rugby athletes who received MAD therapy. Athletes were asked to download the reflex nerve measurement application, Spark, to their smartphones and to set “Numbers” (a minigame in which blue tiles, numbered from 1 to 25, appear randomly on the screen and the player must tap the tiles in numerical order as quickly as possible) as available. They were instructed to take the first measurement of the day 15 min after awakening in a sitting position. 

A second measurement was taken after 30 s. Athletes were instructed to perform three measurements over 5 consecutive days pre- and post-MAD therapy. The measurements were performed in the athletes’ own homes, with consistent living and sleeping conditions, including stretching exercises; however, alcohol consumption was prohibited. Athletes maintained their usual sleep–wake schedules.

### 2.7. Statistical Analyses

Fisher’s one-way analysis of variance was used to compare the OSA severity groups with respect to body composition (age, height, weight, BMI, and neck circumference) and sleep apnea characteristics (REI, ESS, PSQI, and SpO_2 min_). A Bonferroni post hoc correction was applied when significant between-group differences were detected. Paired *t*-tests were performed on REI, SpO_2 min_, and Reaction time, while the Wilcoxon signed-rank test was conducted for ESS at before and after MAD treatment. For all analyses, 95% confidence intervals are presented. All statistical analyses were conducted using SPSS for Windows version 20 (IBM Corp., Armonk, NY, USA). A *p*-value < 0.05 was considered statistically significant.

## 3. Results

### 3.1. Participant Characteristics

The participants’ characteristics are shown in Table 1.

### 3.2. PSQI and ESS Scores

A high proportion of athletes had clinically borderline PSQI and ESS scores. Twenty-nine participants (69.0%) reported poor sleep quality in the past month (PSQI > 5.5). Fourteen participants (33.3%) reported excessive daytime sleepiness (ESS > 10). Twelve participants (28.6%) scored below the cutoff score for both the PSQI and ESS, whereas 14 participants (33.3%) scored above the cutoff score for both the PSQI and ESS (Table 2).

### 3.3. OSA

OSA was diagnosed in 27 (64.3%) of the 42 athletes examined in this study and was classified as mild (*n* = 16, 59.3%), moderate (*n* = 9, 33.3%), or severe (*n* = 2, 7.4%) according to REI severity. Significant differences in the SpO_2 min_ were observed between the different REI severity groups (Table 3).

Athletes with mild, moderate, or severe REI had significantly lower SpO_2 min_ than did those with normal REI (*p* < 0.001). The PSQI and ESS scores did not differ significantly according to REI severity (Table 3).

### 3.4. OSA Severity and Body Composition

Table 4 shows the comparison of age, height, weight, BMI, and neck circumference among the different REI severity groups. A significant difference in age was observed between the normal and severe groups (average, 8.6 years) (*p* = 0.013). Athletes with moderate or severe REI had a significantly larger neck circumference than did those with normal REI (*p* = 0.006) (Table 4).

### 3.5. Sleep Apnea Status and Reaction Time after MAD Therapy

In the subgroup of six athletes who underwent MAD therapy (REI range, 7.2–26.1 times/h), MAD therapy significantly reduced the REI (*p* = 0.028), SpO_2 min_ (*p* < 0.027), and ESS (*p* = 0.026) (Table 5). MAD therapy also significantly reduced the reaction time from 24.2 ± 3.13 s at baseline to 19.7 ± 3.01 s (*p* = 0.026). Paired *t*-tests performed for athletes who played in the forward or back position showed a decrease in the mean reaction time in both groups, although the difference was only significant for athletes who played in the forward position (*p* = 0.018).

## 4. Discussion

Among the 42 elite Japanese rugby union athletes examined in this study, 64.3% had OSA with varying degrees of severity, which was associated with a larger neck circumference. These athletes experienced excessive daytime sleepiness and poor sleep quality, with a short sleep duration (mean, 6.5 h/day). The subgroup of six athletes who underwent MAD therapy exhibited improvements in the REI, SpO_2 min_, ESS, and reaction time.

When analyzing sleep quality, it is important to consider whether sleep duration is sufficient. According to the 2015 NHK Japanese Time Use Survey [29], the average sleep duration was 7 h 27 min for men (*n* = 424) in their 20s. The sleep duration reported in our study suggests that athletes’ sleep duration fell short of that of the age-matched general population. Notably, athletes have a greater need for sleep. Mah et al. [30] reported that, when basketball players were given the opportunity to sleep for an extended period, they had improved shooting accuracy and faster sprint times. Charles and Brent [31] recommended 8–10 h of nocturnal sleep plus a 30-min nap between 2 and 4 pm for athletes. This suggests that the athletes in our study did not meet their sleep needs in relation to their age and athletic demands.

Given the short sleep duration, it was unsurprising that the global PSQI score (>5.5) indicated poor sleep quality in 69.0% of athletes. This contradicts the findings in the general Japanese population, in which a global PSQI score of 4.5 ± 2.1 has been reported for men in their twenties [32]. The study showed that 30.1% of men in their twenties scored above the cutoff PSQI score for poor sleep quality.

In this study, 35.7% of athletes had an ESS score >10, indicating that they experienced high levels of daytime sleepiness. The ESS score was higher than that reported for American footballers (mean ESS, 7.3; ESS score > 10 in >20% of 302 footballers) [33] but was similar to that reported for college basketball players (mean ESS, 9.6 ± 3.8) [34].

In this study, 12 athletes (28.3%) had no sleep problems and did not report excessive daytime sleepiness. The remaining 30 athletes (71.7%) had poor sleep quality or excessive daytime sleepiness. Recently, the effectiveness of sleep hygiene guidance in improving sleep status has been reported [35]. There is a need for the provision of sleep hygiene education, in addition to assessing athletes’ overall training, work, and social schedules, and recovery strategies, with the aim of improving sleep quality.

Athletes who experienced more severe OSA were older than those with normal REI. A recent study [36] showed that older age was associated with increased pharyngeal airway collapsibility during sleep, independent of sex and BMI. This can be explained by either anatomical or neuromuscular changes that occur due to age. Thus, older athletes may be at higher risk, especially considering the characteristic facial morphology of Japanese individuals [23], suggesting that athletes should be monitored for sleep apnea at regular intervals. 

In this study, the BMI increased with increasing REI severity. Itasaka et al. [37] stratified 257 Japanese individuals into three groups according to their BMI (<24.0, 24.0–26.4, and >26.4 kg/m^2^) and reported that those with a higher BMI tended to have higher esophageal pressure and a higher apnea hypoventilation index. These observations support those of Ong and Clerk [38], who reported that sleep-related breathing disorders tended to be more severe among Asians than among Caucasians, despite the lower average BMI. It is noteworthy that the athletes with OSA in our study had a mean neck circumference > 42.8 cm. We also observed a progressive increase in neck circumference (from a mean of 40.2 to 44.2 cm) with increasing REI severity. 

In a previous study [23], the Youden-index-predicted neck circumference cutoff above which the risk of OSA began to increase was determined to be 40.75 cm. In American patients, a neck circumference > 43.0 cm predicted OSA severity with a sensitivity of 67.0% and a specificity of 83.0% [39]. The larger neck circumference observed in our study may be explained by the distinctive facial morphology of Japanese individuals and the size and position of the lower jaw [40], which may be linked to specific genetic traits and/or body dimensions. In general, Japanese individuals have long faces, a racial characteristic associated with increased susceptibility to OSA [41].

The incidence of obesity in Japan was reported to be 28.7% [42], which was lower than that reported in Western populations; however, there was no difference between those who did and did not snore [40]. In sports, such as rugby, body size reduction is not an option due to high-impact physical collisions in the field. Thus, athletes must increase their neck strength, which increases the risk of pharyngeal obstruction and OSA. A large proportion of Japanese rugby athletes in this study were diagnosed with OSA.

The subgroup of six athletes who underwent MAD therapy demonstrated significant improvements in the REI and SpO_2 min_, suggesting that MAD therapy is a viable option for patients with mild-to-moderate OSA with no dental problems. MAD therapy enlarges the velopharyngeal airway, and the reduction in the REI is likely associated with an improvement in the ESS. To date, this is the first reported application of MAD therapy for the treatment of OSA in elite rugby union athletes; however, there have been reports of CPAP therapy for OSA in athletes [21].

Interestingly, MAD therapy improved kinetic vision (the ability to identify moving objects), as measured by reaction time, suggesting that MAD therapy was successful not only in ameliorating SDB, excessive daytime sleepiness, and the REI but also in improving reaction time. We applied a kinetic vision test using a smartphone application that participants can download and then complete on awakening. Previous reports on reaction time have often used the psychomotor vigilance task [43], which takes 10 min to perform and requires the preparation of the test on a computer.

Dinges et al. [44] reported that reducing sleep duration over a week reduced psychomotor vigilance task performance. Poor psychomotor vigilance task performance reflects decreased frontal lobe function [45], with a reduction in executive functioning, such as inhibition and selection. 

Improved reaction time suggests that MAD therapy restores some executive functions by reducing the number of apneic episodes. Future studies should systematically evaluate the impact of poor sleep quality associated with sleep-related breathing disorders on kinetic vision in rugby athletes and other team sports players. This study has some limitations. First, we did not assess the athletes’ objective sleep quality using polysomnography. Second, the subgroup of six athletes who underwent MAD therapy was small; nevertheless, clinically significant findings were observed. 

The number of athletes was small because only a few were willing to commute to the hospital for treatment, as many of the athletes lived far away. In addition, the study did not follow a randomized design with a control group. The lack of a control group for the reaction time test of kinetic vision was another limitation. Furthermore, although measuring reaction time using a smartphone application is convenient and can be performed without training, in the context of sports performance, situational awareness and decision-making could not be evaluated. Physical performance is also important. It will be necessary to combine different methods in the future to evaluate all these factors in a clinical trial.

## 5. Conclusions

In this study, athletes who received MAD treatment exhibited an alleviation of OSA and improvements in subjective measures of sleep quality and measures of reaction time test of kinetic vision. By treating athletes with MAD therapy, dentists can play an important role in identifying sleep-related breathing disorders that may impede sports performance or increase the risk of cardiovascular disease.

## Figures and Tables

**Table 1:** Participant characteristics (*n* = 42).

Age (years)	26.3 ± 3.7
Height (cm)	176.3 ± 5.8
Body weight (kg)	89.5 ± 12.0
BMI (kg/m^2^)	26.7 ± 3.2
Neck circumference (cm)	41.5 ± 2.1
ESS	9.6 ± 3.8
PSQI	6.8 ± 2.3
REI (times/h)	10.8 ± 9.2
SpO_2 min_ (%)	87.4 ± 5.4
Sleep duration (h)	6.5 ± 0.8

Values are expressed as the means ± standard deviations. BMI, body mass index; ESS, Epworth Sleepiness Scale; PSQI, Pittsburgh Sleep Quality Index; REI, Respiratory Event Index; and SpO_2 min_, minimum oxygen saturation.

**Table 2:** PSQI and ESS scores.

PSQI Score	ESS Score		Total
	0.0–10.5	10.5–24.0	
0.0–5.5	12 (28.6)	1 (2.4)	13 (31.0)
>5.5	15 (35.7)	14 (33.3)	29 (69.0)
Total	27 (64.3)	15 (35.7)	42 (100.0)

Values are expressed as n (%). PSQI, Pittsburgh Sleep Quality Index; and ESS, Epworth Sleepiness Scale.

**Table 3:** REI severity and sleep apnea characteristics.

	Normal (*n* = 15)	Mild (*n* = 16)	Moderate (*n* = 9)	Severe (*n* = 2)	F	*p*	Multiple Comparison
ESS	9.7 ± 3.7	9.6 ± 4.3	10.1 ± 4.0	8.0 ± 1.4	0.1	0.935	
PSQI	6.3 ± 2.0	6.7 ± 2.4	7.9 ± 2.4	6.0 ± 2.8	1.0	0.386	
REI (times/h)	2.8 ± 1.0	9.4 ± 2.2	20.6 ± 4.1	37.4 ± 2.1	182.8	<0.001	Normal < mild < moderate < severe
SpO_2 min_ (%)	92.3 ± 2.0	86.1 ± 3.7	84.2 ± 4.4	75.0 ± 1.4	23.9	<0.001	Normal > mild, moderate, severe Mild > severe Moderate > severe

Values are expressed as the means ± standard deviations. REI, Respiratory Event Index; ESS, Epworth Sleepiness Scale; PSQI, Pittsburgh Sleep Quality Index; and SpO_2 min_, minimum oxygen saturation.

**Table 4:** REI severity and body composition.

	Normal (*n* = 15)	Mild (*n* = 16)	Moderate (*n* = 9)	Severe (*n* = 2)	F	*p*	Multiple Comparison
Age (y)	24.9 ± 2.4	26.6 ± 3.9	26.7 ± 3.7	33.5 ± 0.7	4.1	0.013	Normal < severe
Height (cm)	176.3 ± 5.6	176.4 ± 5.7	175.2 ± 6.3	180.5 ± 9.2	0.4	0.730	
Body weight (kg)	85.0 ± 11.1	89.8 ± 12.0	94.1 ± 12.5	100.4 ± 3.4	1.8	0.164	
BMI (kg/m^2^)	27.3 ± 2.1	28.8 ± 3.1	30.6 ± 3.9	31.0 ± 4.2	2.8	0.053	
Neck circumference (cm)	40.2 ± 1.6	41.7 ± 1.6	42.6 ± 2.6	44.2 ± 0.6	4.9	0.006	Normal < moderate, severe

Values are expressed as the means ± standard deviations. REI, Respiratory Event Index; and BMI, body mass index.

**Table 5:** Sleep apnea status before and after MAD therapy (*n* = 6).

	Before MAD Therapy	After MAD Therapy	*p*
REI (times/h)	17.2 ± 8.3	5.6 ± 3.2	0.028
SpO_2 min_ (%)	82.3 ± 3.3	91.2 ± 2.0	0.027
ESS	13.7 ± 2.0	8.8 ± 1.7	0.026

Values are expressed as the means ± standard deviations. MAD, mandibular advancement device; REI, Respiratory Event Index; SpO_2 min_, minimum oxygen saturation; and ESS, Epworth Sleepiness Scale.

## Data Availability

The data that support the findings of this study are available from the corresponding author (H.S.) upon reasonable request.
